# SENP1 modulates microglia‐mediated neuroinflammation toward intermittent hypoxia‐induced cognitive decline through the de‐SUMOylation of NEMO

**DOI:** 10.1111/jcmm.16689

**Published:** 2021-06-13

**Authors:** Hongwei Wang, Tianyun Yang, Jinyuan Sun, Sisen Zhang, Song Liu

**Affiliations:** ^1^ The Second School of Clinical Medicine Southern Medical University Guangzhou China; ^2^ Department of Respiratory Medicine Xinhua Hospital School of Medicine Shanghai Jiao Tong University Shanghai China; ^3^ Affiliated Zhengzhou People's Hospital, The Second School of Clinical Medicine, Southern Medical University Zhengzhou China

**Keywords:** cognitive dysfunction, intermittent hypoxia, microglia, NEMO, neuroinflammation, SENP1

## Abstract

Intermittent hypoxia (IH)‐induced cognition decline is related to the neuroinflammation in microglia. SUMOylation is associated with multiple human diseases, which can be reversed by sentrin/SUMO‐specific proteases 1 (SENP1). Herein, we investigated the role of SENP1 in IH‐induced inflammation and cognition decline. BV‐2 microglial cells and mice were used for inflammatory response and cognition function evaluation following IH treatment. Biochemical analysis and Morris water maze methods were used to elaborate the mechanism of SENP1 in IH impairment. Molecular results revealed that IH induced the inflammatory response, as evidenced by the up‐regulation of NF‐κB activation, IL‐1β and TNF‐α in vitro and in vivo. Moreover, IH decreased the expression of SENP1, and increased the SUMOylation of NEMO, not NF‐κB P65. Moreover, SENP1 overexpression inhibited IH‐induced inflammatory response and SUMOylation of NEMO. However, the inhibitions were abolished by siRNA‐NEMO. In contrast, SENP1 depletion enhanced IH‐induced inflammatory response and SUMOylation of NEMO, accompanying with increased latency and reduced dwell time in mice. Overall, the results demonstrated that SENP1 regulated IH‐induced neuroinflammation by modulating the SUMOylation of NEMO, thus activating the NF‐κB pathway, revealing that targeting SENP1 in microglia may represent a novel therapeutic strategy for IH‐induced cognitive decline.

## 1 INTRODUCTION

Repeated episodes of upper airway obstruction in obstructive sleep apnoea (OSA), a process named intermittent hypoxia (IH), which induced neuroinflammation in microglia, is highly associated with cognitive decline.^1^, ^2^, ^3^, ^4^ Microglia, the resident immune cell in central nervous system (CNS), constitute the first line of defence against brain injury.^5^ Studies have shown that microglia‐mediated inflammation plays a crucial role in IH‐induced cognitive decline.^2^, ^6^

Evidence on the precise mechanism underlying the neuroinflammation in IH impairment and the resultant cognitive decline has indicated that the mechanism may involve the inhibitory kappa B kinase (IκK)/inhibitory kappa B (IκB)/nuclear factor kappa B (NF‐κB) pathway.^7^, ^8^ In normal state, NF‐κB is located in cytoplasm conjugated to IκB, and it can translocate into the cell nucleus after being activated and promote the expression of proinflammatory genes.^9^, ^10^ Actually, the relationship between NF‐κB and the release of inflammatory factors induced by IH indicated that NF‐κB participated in the inflammatory injury of cardiovascular disease caused by OSA.^11^ Moreover, our previous study showed that the activation of NF‐κB pathway is implicated in IH‐induced inflammatory response in BV‐2 microglial cells.^12^, ^13^ Despite these, the further specific mechanism underlying the activation of NF‐κB pathway upon IH‐induced inflammatory response remains unclear.

Small ubiquitin‐like modifier (SUMO) is an essential post‐translational modification, named SUMOylation, characterized by addition or detachment of SUMO proteins to lysine residues on target proteins, altering their subcellular localization, activity and stability.^14^ Evidence has demonstrated that SUMOylation participates in the NF‐κB pathway, playing an essential regulatory role in the inflammation and the secretion of various chemokines.^15^, ^16^ Almost all activators of NF‐κB depend on the release of IκB through activating an IκB kinase (IKK) complex that induces IκB degradation, allowing freed NF‐κB to translocate into the nucleus and activate the transcription of target genes. NEMO, an essential regulatory scaffolding subunit of IKK complex, indirectly regulates the activity of NF‐κB.^17^ SUMOylated NEMO is distributed in the nucleus and could transport into cytoplasm, leading to the activation of IKK and NF‐κB in response to stimuli.^17^ Therefore, SUMOylation and de‐SUMOylation exerts an essential role in regulating the activation of NF‐κB and NF‐κB‐dependent transcription.^18^ SUMOylation is a dynamic process that can be reversed by SUMO‐specific proteases (SENPs). Among the SENPs (SENP1~3 and SENP5~7) (31), SENP1 has been reported to involve in various human cancers.^19^, ^20^, ^21^ Accumulating evidence has demonstrated that SENP1 can deconjugate a number of SUMOylated proteins, including HIF‐1α,^22^ UBE2T ^23^ and Akt.^24^ Our previous study tested the six SENPs, only the expression of SENP1 showed a significant reduction in microglial cells in response to IH, and SENP1 overexpression attenuated IH‐induced effects on microglia.^25^ Moreover, one study focussing on diabetic nephropathy study revealed that SENP1 depletion enhanced the SUMOylation of NEMO, resulting in the inflammatory response through activating the NF‐κB pathway.^26^ However, the mechanism of SUMOylation and de‐SUMOylation of NEMO in IH‐associated NF‐κB pathway remains unknown. Based on these findings, we hypothesized that SENP1 regulates microglia‐mediated neuroinflammatory response toward IH‐induced cognitive decline through the de‐SUMOylating of NEMO and subsequently inhibiting the activation of NF‐κB.

In the present study, we determined whether SENP1 plays important roles in IH‐induced cognitive dysfunction by affecting microglia‐mediated inflammatory response in the hippocampus through the de‐SUMOylating of NEMO using an IH model in vitro and in vivo.

## 2 MATERIALS AND METHODS

### 2.1 Transcriptional analysis of SUMOylation protein

Differentially expression genes (DEGs) were selected on the basis of a false discovery rate value <0.05 from three data sets, including GSE8262,^27^ GSE62385 ^28^ and GSE1357,^29^ and related to SUMOylation from the Human Protein Atlas. The DEGs were required to meet three inclusion criteria: (a) essential and distinct expression in the hippocampus, (b) involvement in the SUMOylation process and (c) association with hippocampal hypoxia injury. The bioinformatics analysis was performed with R software (version 4.0.3 GUI 1.73, Catalina build).

### 2.2 Cell culture

BV‐2 microglial cells were obtained from Prof. Zeng‐qiang Yuan (Chinese Academy of Sciences) and were cultured as described in previous report.^25^ After IH treatment, the supernatant medium of both normoxia and IH groups was collected for subsequent experiments.

### 2.3 IH treatment of cells

BV‐2 cells were placed in an IH culture chamber, and the chamber alternated the oxygen levels between 1% and 21% within 400 s, in a cyclic repetitive format for 12 h. The total time of O2 concentration decreased from 21% to 1% and lasted at the 1% level was 200 s; the total time of O2 concentration increased from 1% to 21% and lasted at the 21% level was also 200 s. The control group was cultured in a 21% oxygen content state.

### 2.4 SENP1 adenovirus transfection

SENP1 adenovirus or negative control adenovirus solution was added into the cell medium at an 80% cell density. The medium was then removed and replaced with a fresh medium after 8 h. qRT‐PCR, Western blot analysis and immunofluorescent staining were used to detect the transfection efficiency.

### 2.5 Small interfering RNA (siRNA) transfection

NEMO expression in BV‐2 cells was knocked down using siRNA, and siRNA‐NT served as the negative control (Santa Cruz). siRNA was transfected into BV‐2 cells with SENP1 overexpressed using Lipofectamine 3000 reagent (Invitrogen). The transfection efficiency was detected by qRT‐PCR and Western blot analysis.

### 2.6 Animals

C57BL/6J mice, aged 10‐12 weeks (weight, 25 ± 4 g), were obtained from the Department of Experimental Animal Science, the School of Medicine, Shanghai Jiao Tong University. Mice were maintained following guidelines published by the US National Institutes of Health. All experimental procedures were conducted in accordance with the National Institutes of Health Guide for the Care and Use of Laboratory Animals and approved by the ethics committee (App. No.: XHEC‐F‐2019‐030).

#### 2.6.1 Generation of SENP1^±^ mice

The SENP1^±^ ES cell line was obtained from BayGenomics. ES cells were generated using a gene trap protocol with the trapping construct pGT1Lxf containing the intron from the engrailed‐2 gene upstream of the gene encoding the β‐galactosidase/neomycin‐resistance fusion protein. The vector was inserted into intron of the SENP1 locus. A male chimeric mouse was generated from the ES cell line.^22^ SENP1 knockdown (SENP1^±^) mice were generated by mating the chimeric male and SENP1*^+/+^* female mice. An equal number of male and female mice were included in each group to avoid any potential sex‐related effect. SENP1*^+/+^* and SENP1^±^ mice were randomly divided into normoxia and IH groups, respectively, yielding four groups (n = 12 per group). Mice genotype identification was performed before the experiment.

### 2.7 IH treatment of mice

Mice from the normoxia and IH groups were placed in two identical commercially designed chambers (Bio‐instruments, NY). The chambers were connected to supplies of pure O_2_ and N_2_ to produce changes in O_2_ concentration. During the exposure periods, O_2_ concentration was cyclically reduced from 21% to 10% in the IH group. The mice were left in the chambers for 4 weeks and exposed to intermittent hypoxia (IH group) or normoxic conditions (normoxia group) for a total of 8 h a day. Sodasorb CO_2_ absorbent was used to remove the excess of CO_2_.

The IH protocol consisted of 4‐min cycles, including 2 min of hypoxia (10% O_2_) and 2 min of normoxia (21% O_2_). Hypoxia was initiated by injecting N_2_ into the chamber, resulting in a low point of 10% O_2_ in 2 min, after which O_2_ was injected over 2min, resulting in 21% O_2_ within 2 min. The normoxia group mice were exposed to normoxic conditions in a chamber identical to the IH group. For the remaining 16 h, O_2_ concentration was kept at 21% in both IH and normoxia groups.

### 2.8 Morris water maze (MWM) test

Spatial learning and memory were assessed by the MWM test as previously described.^30^ Briefly, the acquisition test was performed four times per day for five consecutive days. The time to find the hidden platform was defined as the escape latency. Retention tests were performed one day after the final acquisition session. The platform was removed, mice were allowed to explore freely for 60 s. The time required and the number of crossings over the platform area were recorded. The entire experiment was recorded using a digital camera trace monitoring system.

### 2.9 Animal sample collection

Mice were anaesthetized with 5% pentobarbital sodium and then killed to collect whole brains or hippocampus for cardiac saline perfusion. Brain samples were fixed with 4% paraformaldehyde for immunohistochemistry, and hippocampus samples were stored at −80℃ for molecular biological analysis.

### 2.10 Quantitative reverse transcription‐polymerase chain reaction

Quantitative reverse transcription‐polymerase chain reaction (qRT‐PCR) was performed as described previously.^31^ Mouse primers used are listed: SENP1 forward: AGTAAAGAAGGTTCCGGTTCCCG, reverse: GCCGCCACTCACCGAAC; NF‐κB p65 forward: TGGGGGTACGGGTGAATCTT, reverse: CATAGTAGCCATCCCGGCAG; IκB‐α forward: GTGACGCAAGACGTAGAGGAA, reverse: GGAGTGAAGCAACCACATGC; NEMO forward: CTTGTTTTGGCTCAGCCTGC, reverse: GCTGGAGGGTCTCAGGAGTA; GAPDH forward: AGGTCGGTGTGAACGGATTTG, reverse: TGTAGACCATGTAGTTGAGGTCA. In brief, total RNA was extracted using RNAiso Plus reagent, reverse‐transcribed to complementary DNA and then amplified using an SYBR Green Master Mix kit (Takara). Each independent experiment was repeated three times. The mRNA levels were calculated relative to GAPDH using the 2^−ΔΔCT^ method.

### 2.11 Western blot analysis

Total protein of cells and hippocampus samples was extracted using RIPA solution. Denatured protein was separated by sodium dodecyl sulphate‐polyacrylamide gel electrophoresis and then transferred onto nitrocellulose membranes (Millipore, USA), which were blocked and then incubated with SENP1 (1:1000, ab236094, Abcam), SUMO‐1 (1:1000, sc‐5308, SANTA), NF‐κB p65 (1:1000, ab239882, Abcam), IκB‐α (1:1000, ab32518, Abcam), NEMO (1:1000, ab178872, Abcam), IL‐1β (1:1000, ab234437, Abcam), TNF‐α (1:1000, ab215188, Abcam) and β‐actin (1:1000, ab179467, Abcam) primary antibodies at 4° overnight. The membranes then were further incubated with secondary antibodies. The bands were visualized using the enhanced chemiluminescence system (Millipore). The bands’ density was quantified with ImageJ software (NCIH) and normalized to that of β‐actin.

### 2.12 Co‐immunoprecipitation assay

Cells or fresh hippocampus samples were lysed with ice‐cold cell lysis buffer, and supernatant was collected. The experimental procedure for the determination of protein SUMOylation is briefly described as follows: after pre‐clearing the lysate supernatant with protein A/G PLUS‐agarose, anti‐NEMO and protein A/G PLUS‐agarose were added to the lysate and cultured at 4℃ overnight. The resulting immune complexes were washed with 2× loading buffer. Finally, the complex was subjected to Western blot analysis using specific antibodies to detect the SUMOylation of proteins.

### 2.13 Immunohistochemical analysis

Brain tissue was dehydrated, embedded and sectioned, and the section was deparaffinized and rehydrated, after which antigen retrieval was achieved in a microwave for 15 min. Subsequently, the section was blocked and incubated with primary antibodies (SENP1) at 4℃ overnight. The section was then incubated with an appropriate HRP‐conjugated secondary antibody and counterstained with haematoxylin.

### 2.14 Immunofluorescence analysis in vitro

The cells were collected, permeabilized, blocked, incubated overnight with SENP1 primary antibody (1:100, ab236094, Abcam) and then incubated with Alexa Fluor 647 conjugated anti‐mouse IgG (1:100, 150115, Abcam) for 1 h. Finally, cell nuclear staining was stained using 4′,6‐diamidino‐2‐phenylindole (DAPI). Images were observed and captured with a fluorescence microscope (Olympus, Japan).

### 2.15 ELISA assay

Levels of IL‐1β and TNF‐α in supernatants were measured by ELISA kits (BioLegend) following the manufacturers’ instructions.

### 2.16 Statistical analysis

All statistical analyses were performed using Statistical Package for Social Sciences (version 26.0, Chicago, USA) software. Quantitative data are presented as the mean ± standard deviation (SD). Differences between experimental and control groups were determined by Student's *t* test. Repeated measures analysis of variance (ANOVA) was performed for the water maze data as appropriate. *P* < .05 indicated statistical significance.

## 3 RESULTS

### 3.1 Expression profiling identifies SENP1 in hippocampus as a potential protective factor in IH impairment

**FIGURE 1:**
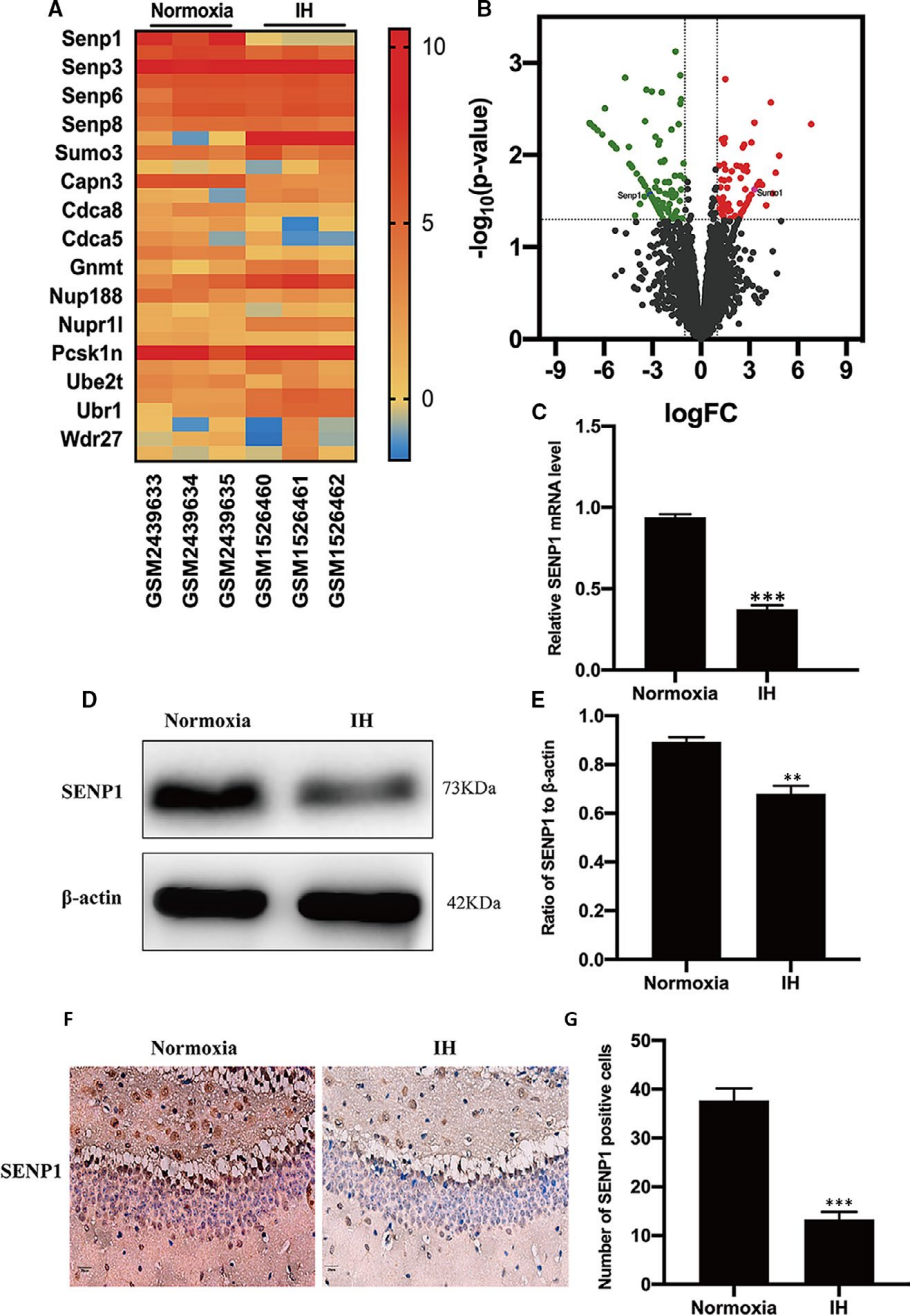
SENP1 is down‐regulated in the hippocampus of mice with intermittent hypoxia (IH) treatment. (A) Heat map of mRNA showing the differences in SENP1 expression between the normoxia and IH groups. ‘Red’ indicates up‐regulation and ‘Blue’ indicates down‐regulation. (B) Volcano map of differentially expressed genes in hippocampus. The abscissa represents log [fold‐change (FC)], and the ordinate represents log_10_ (p value). ‘Red’ dots represent up‐regulated genes, whereas ‘green’ dots represent down‐regulated genes in the hippocampus of mice with IH treatment. (C) qRT‐PCR, (D, E) Western blot analysis and (F, G) immunohistochemical assay showed the differences in the expressions of SENP1 between the normoxia and IH groups. n = 3 per group. ^**^ *P* < .01, ^***^ *P* < .001 versus the normoxia group. Scale bar = 20 μm. SENP1, small ubiquitin‐related modifier protein‐specific protease 1; qRT‐PCR, quantitative reverse transcriptase polymerase chain reaction; Normoxia: mice were treated under normoxia condition; IH: mice were treated under IH condition

According to bioinformatics analysis, the expression of SENP1 in the hippocampus of mice with IH treatment decreased compared to that with normoxia treatment (Figure 1A). The volcano map also showed that SENP1 was down‐regulated while SUMO‐1 was up‐regulated in mice's hippocampus with IH treatment (Figure 1B). Moreover, qRT‐PCR (Figure 1C), Western blot analysis (Figure 1D, E) and immunohistochemical analysis (Figure 1F, G) results also revealed that the expression of SENP1 was significantly decreased in hippocampus of mice with IH treatment compared to that with normoxia treatment, suggesting that SENP1 in hippocampus may play an essential role in IH impairment.

### 3.2 IH triggers inflammatory response in vitro

**FIGURE 2:**
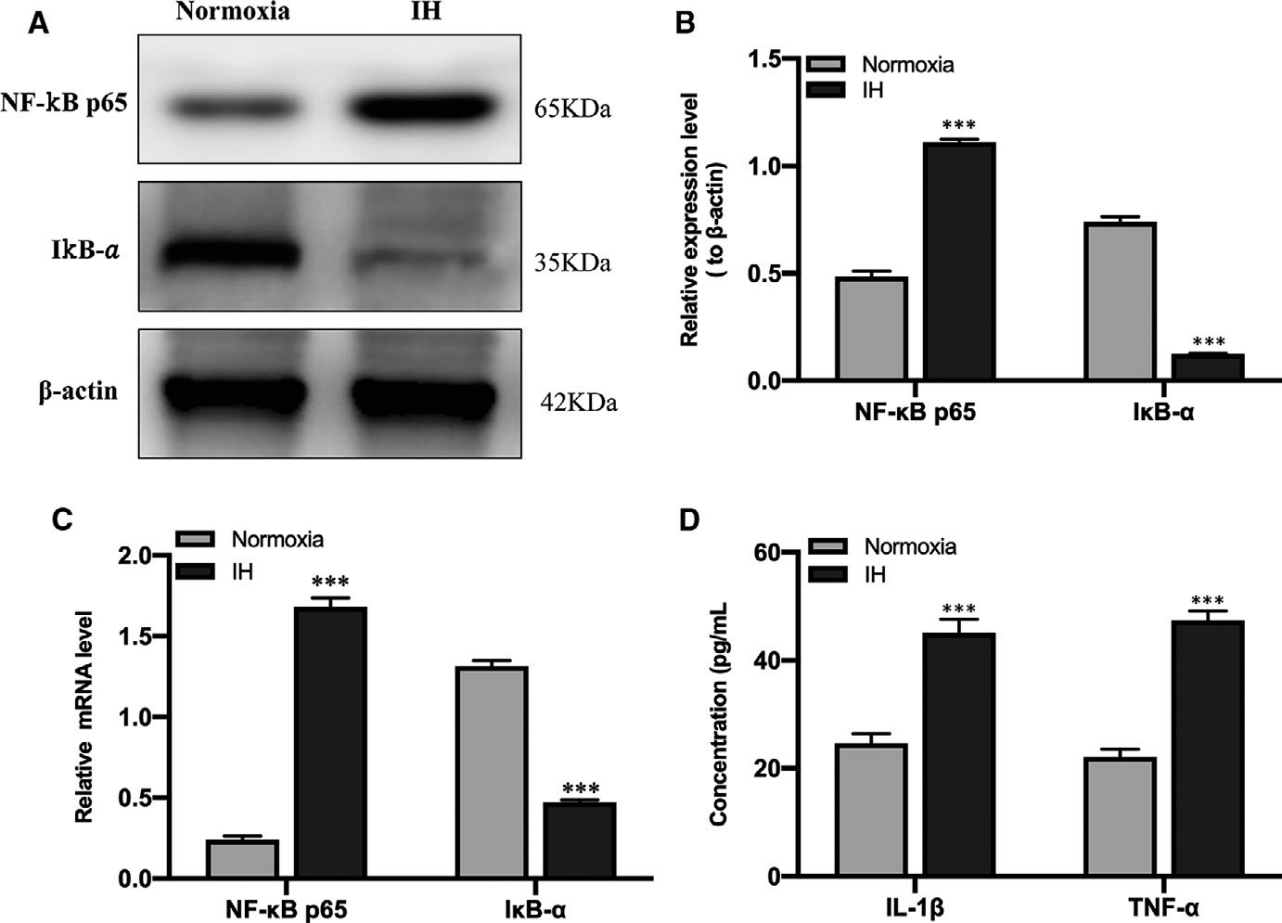
IH‐induced the activation of NF‐κB and the up‐regulation of proinflammatory cytokines. (A, B) The expression of NF‐κB p65 and IκB‐α in BV‐2 cells under normoxia and IH conditions was detected by Western blot analysis. n = 3 per group. ^***^ *P* < .001 versus the normoxia group. (C) The qRT‐PCR showed the expression of NF‐κB p65 and IκB‐α in BV‐2 cells under normoxia and IH conditions. n = 3 per group. ^***^ *P* < .001 versus the normoxia group. (D) The expression of IL‐1β and TNF‐α in BV‐2 cells under normoxia and IH conditions was detected by ELISA. n = 3 per group. ^***^ *P* < .001 versus the normoxia group. Normoxia: BV‐2 cells were cultured in normoxia condition; IH: BV‐2 cells were exposed to IH. ELISA, enzyme‐linked immunosorbent assay; NF‐κB, nuclear factor kappa B; IL‐1β, interleukin‐1β; TNF‐α, tumour necrosis factor‐α

After IH treatment, the expressions of NF‐κB p65 and IκB‐α in BV‐2 cells were evaluated by Western blot analysis and qRT‐PCR. Western blot analysis results showed that the expression of NF‐κB p65 was significantly up‐regulated, while IκB‐α was significantly down‐regulated in the IH group compared to the normoxia group (Figure 2A,B); qRT‐PCR results showed the same trend alteration (Figure 2C), which represent the activity of NF‐κB. Moreover, the ELISA results showed that the expression of IL‐1β and TNF‐α was significantly increased after IH treatment (Figure 2D), indicating that IH induced inflammatory response in vitro.

### 3.3 SENP1 overexpression attenuates inflammatory response

**FIGURE 3:**
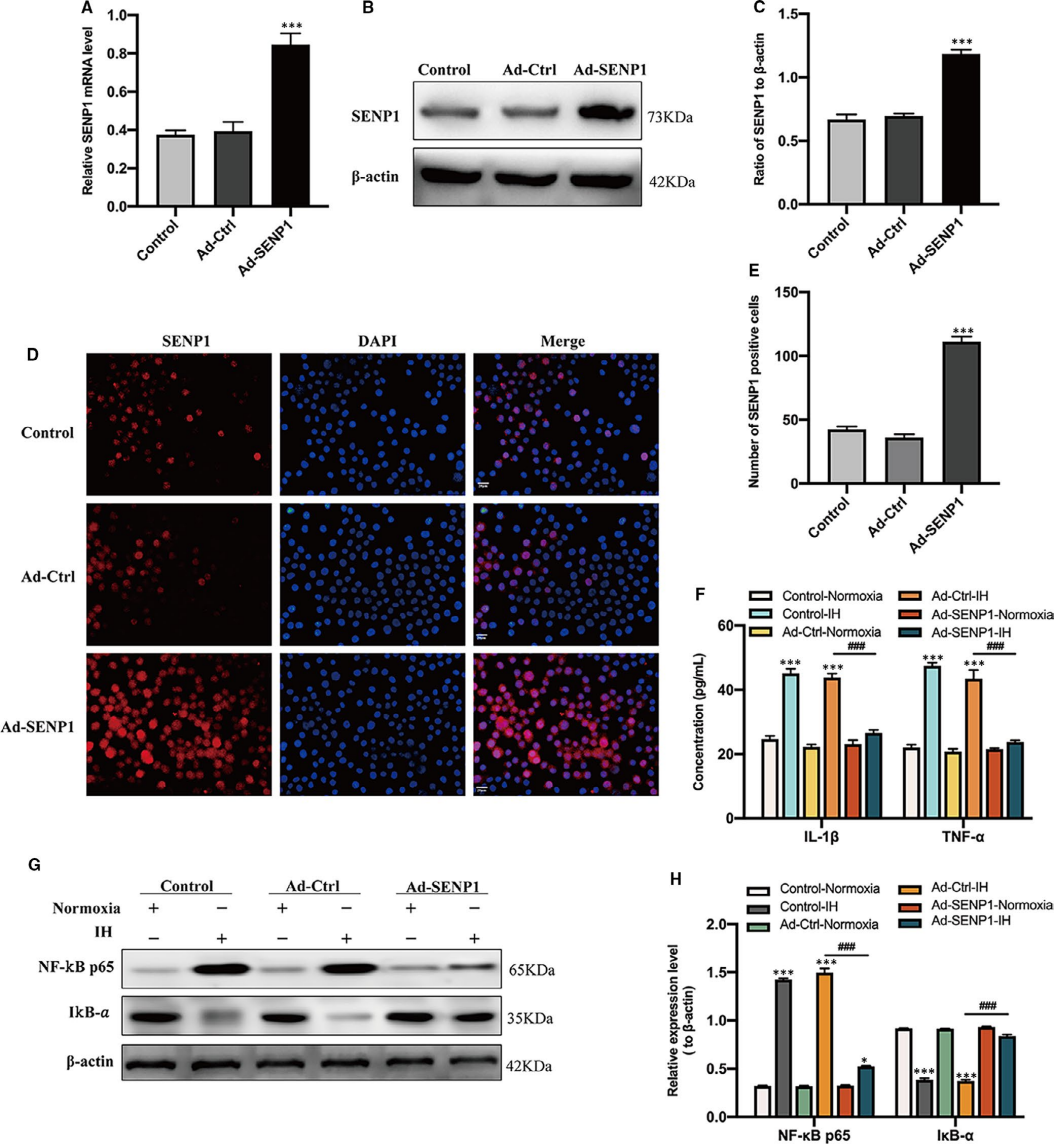
Overexpression of SENP1 inhibits IH‐induced activation of NF‐κB and the up‐regulation of proinflammatory cytokines in BV‐2 cells. (A) qRT‐PCR, (B, C) Western blot analysis and (D, E) cellular immunofluorescence assay showed the overexpression of SENP1 in BV‐2 cells after transfection. Untreated BV‐2 cells were used as controls. n = 3 per group. ^***^ *P* < .001 versus the Ad‐Ctrl group. n = 3 per group. Scale bar = 20 μm. (F) The expression of IL‐1β and TNF‐α in BV‐2 cells with SENP1 overexpression under normoxia and IH conditions was detected by ELISA. n = 3 per group. ^***^ *P* < .001 versus the normoxia group; **^###^** *P* < .001 versus the Ad‐Ctrl group under IH condition. (G, H) The expression of NF‐κB p65 and IκB‐α was detected by Western blot analysis. n = 3 per group. ^*^ *P* < .05, ^***^ *P* < .001 versus the normoxia group; **^###^** *P* < .001 versus the Ad‐Ctrl group under IH condition

After adenovirus transfection, the expression of SENP1 in BV‐2 cells was significantly up‐regulated compared to the control group, which was confirmed by qRT‐PCR (Figure 3A), Western blot analysis (Figure 3B,C) and cellular immunofluorescence analysis (Figure 3D,E). Next, the expression of NF‐κB p65 and IκB‐α was detected using Western blot analysis, and the expression of IL‐1β and TNF‐α was examined by ELISA. There is no significant difference in NF‐κB p65 and IκB‐α expression under normoxia condition, while SENP1 overexpression down‐regulated the expression of NF‐κB p65 and up‐regulated the expression of IκB‐α under IH condition (Figure 3G,H). The expression of IL‐1β and TNF‐α also revealed a similar trend: no significant difference under normoxia condition and significant inhibition of the IH‐induced up‐regulation (Figure 3F). Taken together, SENP1 overexpression inhibited the inflammatory response with the de‐activation of NF‐κB and down‐regulation of inflammatory cytokines.

### 3.4 IH induces the SUMOylation of NEMO

**FIGURE 4:**
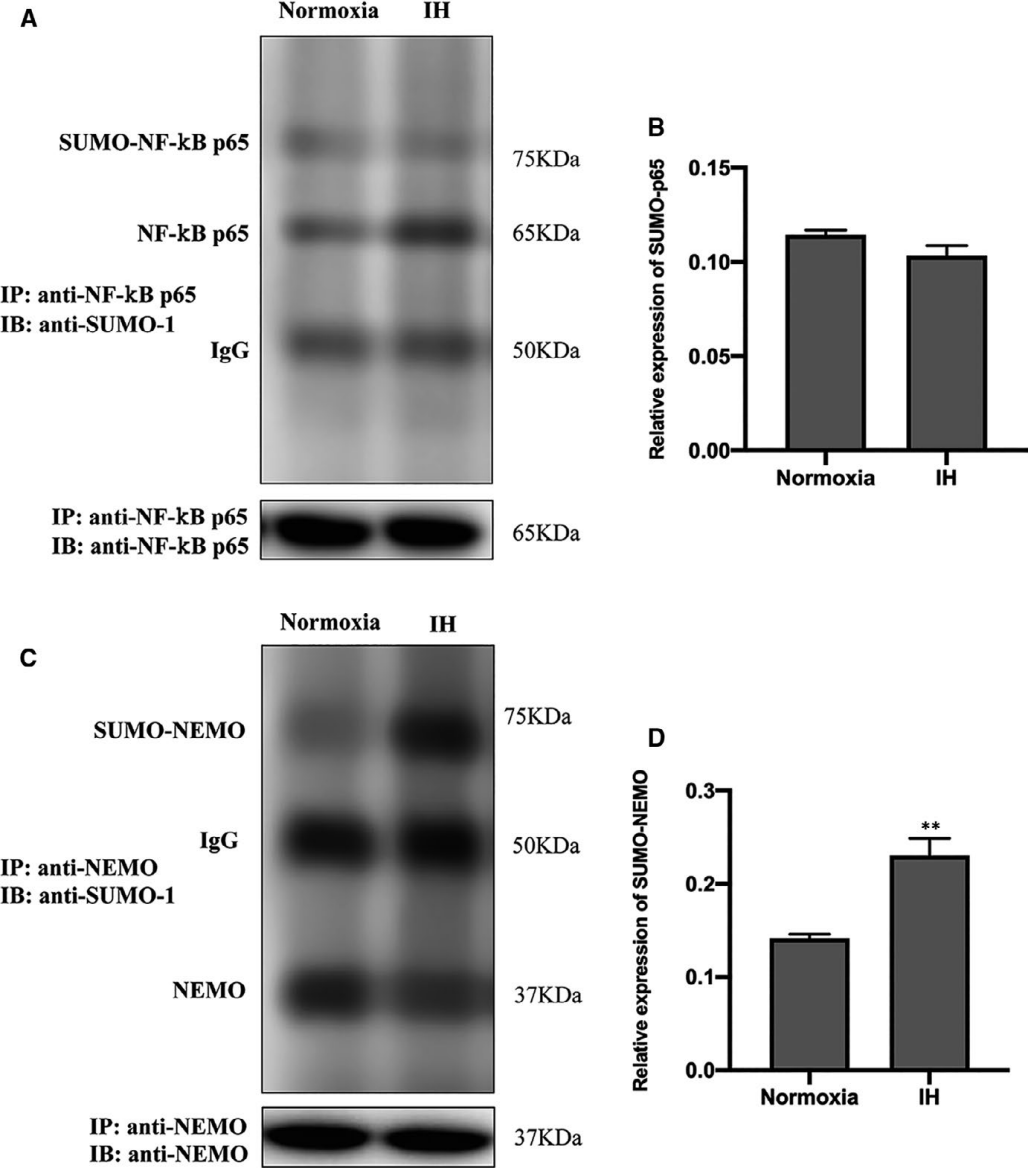
The influence of IH on the SUMOylation of NF‐κB p65 and NEMO. (A, B) The effects of IH on the SUMOylation of NF‐κB p65 and (C, D) NEMO were detected by co‐immunoprecipitation (CO‐IP) followed by Western blot analysis. ^**^ *P* < .01 versus the normoxia group; NEMO, NF‐κB essential modulator; SUMO, small ubiquitin‐like modifier

The SUMOylation of NEMO and NF‐κB p65 was estimated by co‐immunoprecipitation (CO‐IP) followed by Western blot analysis. No significant difference was observed in NF‐κB p65 (Figure 4A,B). However, the SUMOylation of NEMO was significantly increased in IH group compared to the normoxia group (Figure 4C,D). These results were consistent with the hypothesis above that IH induces the SUMOylation of NEMO.

### 3.5 SENP1 overexpression attenuates inflammatory response by inhibiting the SUMOylation of NEMO

**FIGURE 5:**
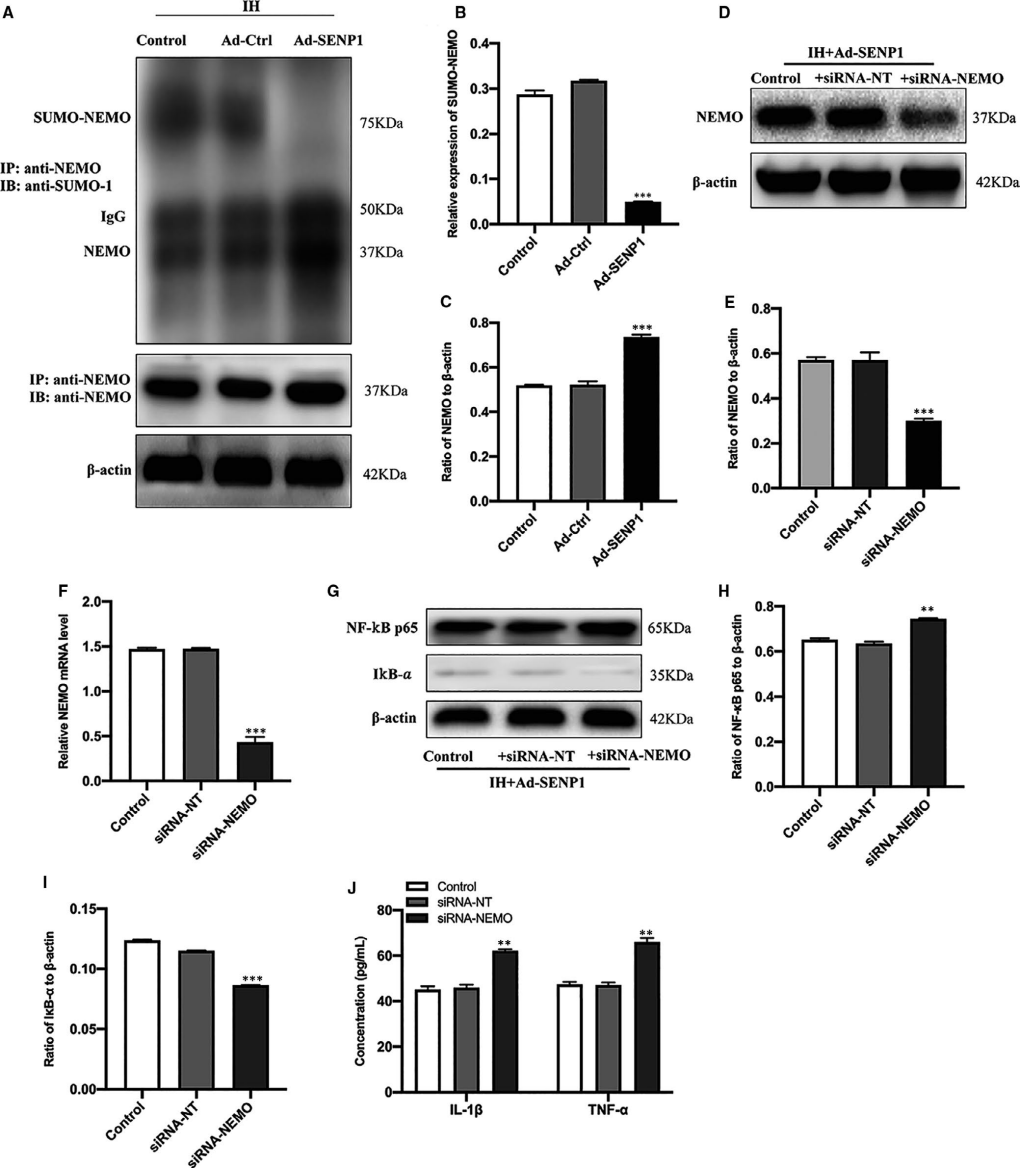
Overexpression of SENP1 attenuates IH‐induced inflammatory response by inhibiting the SUMOylation of NEMO in BV‐2 cells. (A, B) The effects of SENP1 overexpression on IH‐induced SUMOylation enhancement of NEMO. (A, C) SENP1 overexpression elevated the levels of NEMO in IH‐treated BV‐2 cells. ^***^ *P* < .001 versus the Ad‐Ctrl group. (D–F) BV‐2 cells were transfected with siRNA‐NEMO or with siRNA‐NT as the blank control. Untreated BV‐2 cells were used as the control. The siRNA‐mediated transfection efficiency was determined by (D, E) western blot analysis and (F) qRT‐PCR. n = 3 per group. ^***^ *P* < .001 versus siRNA‐NT. (G–I) The expression of NF‐κB p65 and IκB‐α in BV‐2 cells with SENP1 overexpression under IH condition was detected by western blot analysis. n = 3 per group. ^**^ *P* < .01, ^***^ *P* < .001 versus the siRNA‐NT group. (J) The expression of IL‐1β and TNF‐α was detected by ELISA. n = 3 per group. ^**^ *P* < .01 versus the siRNA‐NT group

To reveal the regulatory mechanism of SENP1 on IH‐induced inflammatory response, we evaluated the effect of SENP1 overexpression on the SUMOylation of NEMO by CO‐IP in SENP1 overexpression group and control group under IH condition in vitro. The results revealed that SENP1 overexpression significantly inhibited the SUMOylation of NEMO (Figure 5A,B) and elevated the level of NEMO (Figure 5A,C) compared to the control group under IH condition, and suggested that SENP1 overexpression elevated the level of NEMO in BV‐2 cells and inhibited the SUMOylation of NEMO induced by IH. Subsequently, to determine the effect of NEMO on IH‐induced inflammatory response, the expression of NEMO in SENP1‐overexpressing BV‐2 cells was interrupted by siRNA‐NEMO. The Western blot analysis and qRT‐PCR results showed that the expression of NEMO in the siRNA‐NEMO group was significantly decreased compared with the blank control group after gene silencing, and no significant difference between the negative silenced group and the control group (Figure 5D–F). Then, the expression of NF‐κB p65 and IκB‐α was detected using Western blot analysis, and the expression level of IL‐1β and TNF‐α was examined by ELISA. Western blot analysis results showed that NF‐κB p65 was significantly up‐regulated and IκB‐α was significantly down‐regulated in the siRNA‐NEMO group compared to the negative silenced group (Figure 5G–I). The ELISA results revealed that IL‐1β and TNF‐α were significantly increased in the siRNA‐NEMO group compared to the negative silenced group (Figure 5J). These results implied that SENP1 attenuates IH‐induced inflammatory response by inhibiting the SUMOylation of NEMO in BV‐2 cells.

### 3.6 Deletion of SENP1 accelerates IH‐induced inflammatory response by promoting the SUMOylation of NEMO in vivo

**FIGURE 6:**
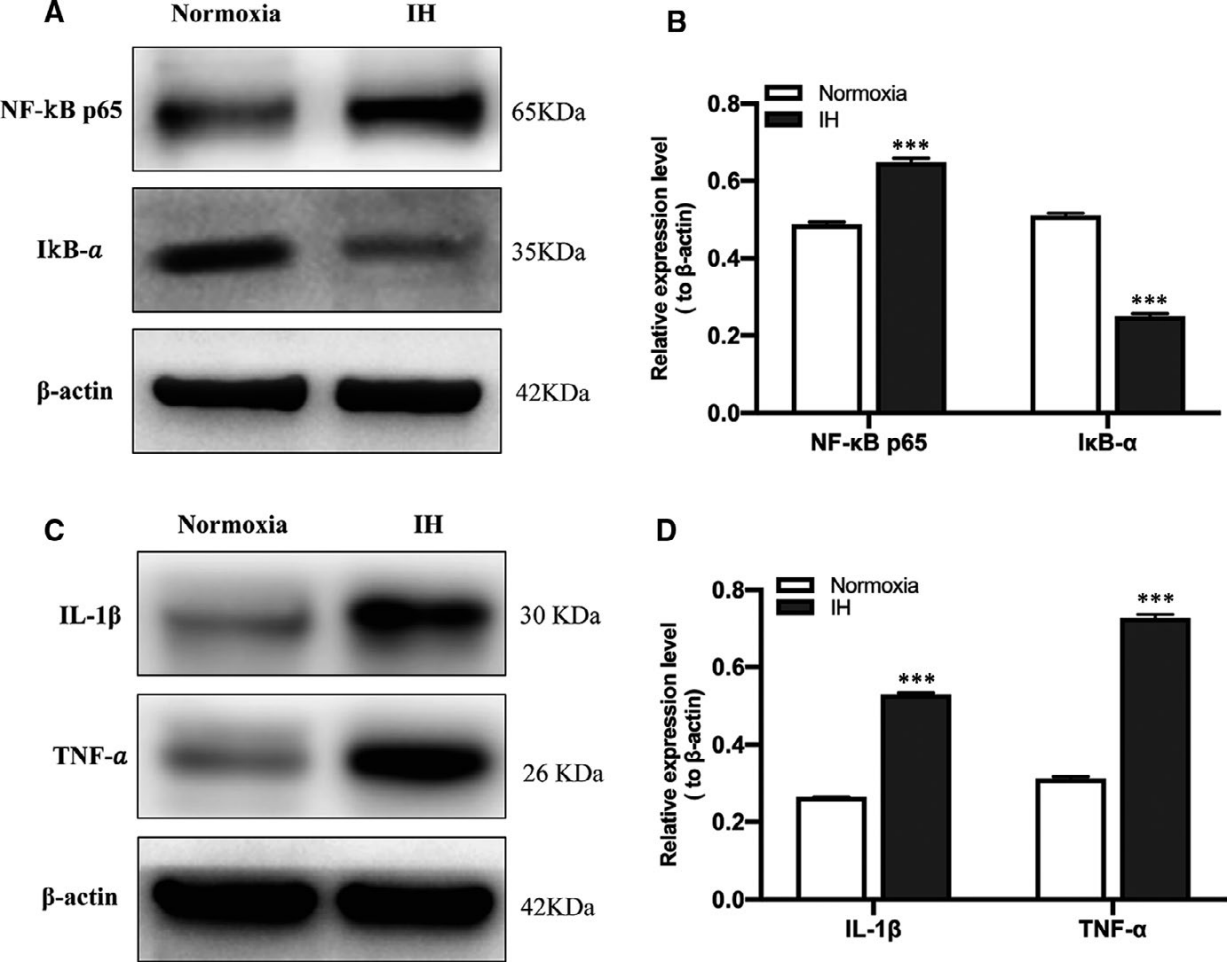
Intermittent hypoxia induced the activation of NF‐κB and up‐regulation of proinflammatory cytokines in vivo. (A–D) The expression of NF‐κB p65, IκB‐α (A, B), IL‐1β and TNF‐α (C, D) in the hippocampus of mice with normoxia and IH treatment was detected by Western blot analysis. n = 3 per group. ^***^ *P* < .001 versus the normoxia group

**FIGURE 7:**
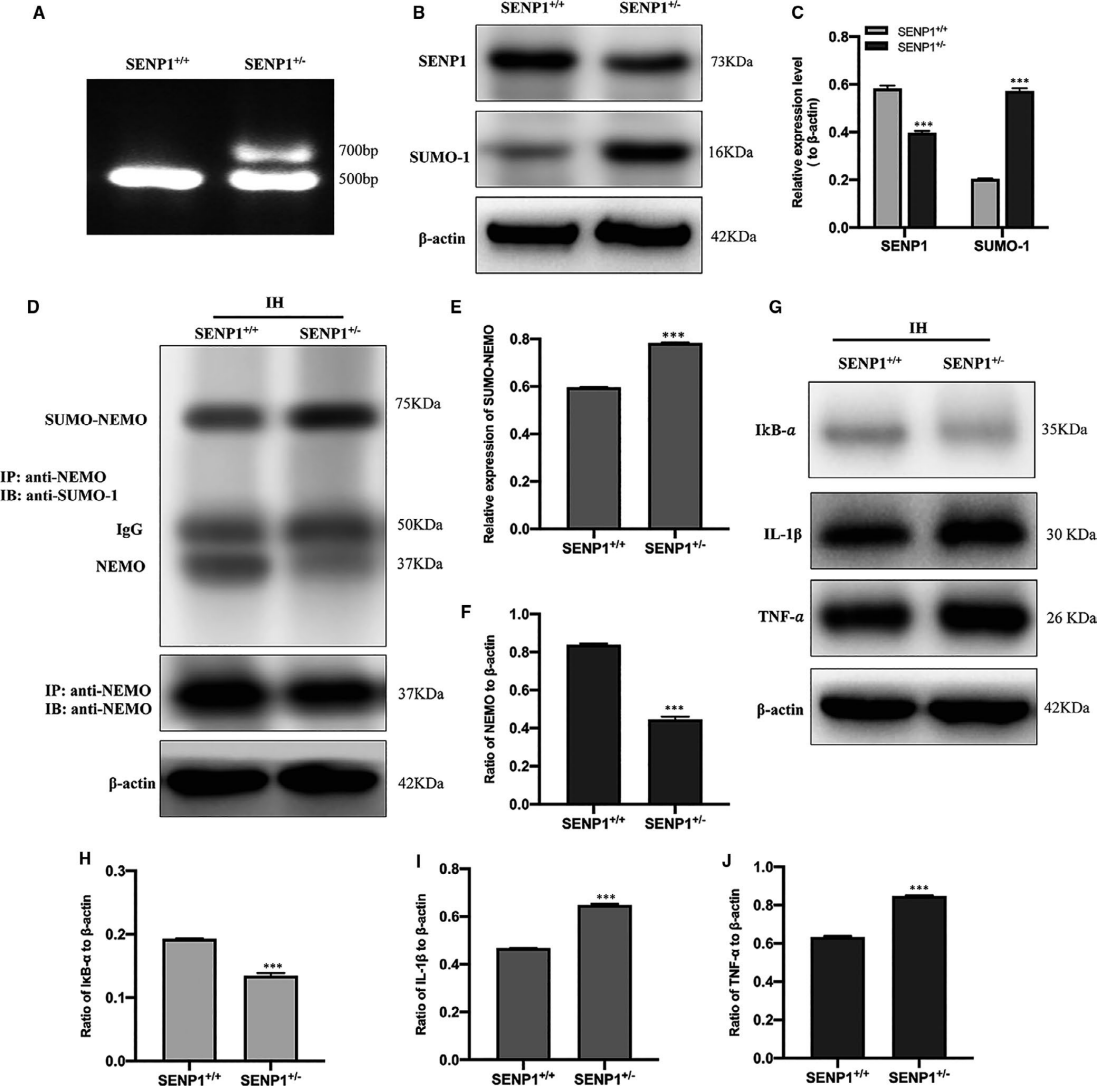
SENP1 depletion accelerates IH‐induced inflammatory response by promoting the SUMOylation of NEMO in vivo. (A) Agarose gel electrophoresis identification of mouse genotype. SENP1^+/+^, represents the homozygote of wild‐type mouse; SENP1^±^, represents mutant heterozygote of SENP1 knockdown mice. (B, C) The expression of SENP1 and SUMO‐1 in the hippocampus of mice with or without SENP1 knockdown was detected by Western blot analysis. n = 3 per group. ^***^ *P* < .001 versus the SENP1^+/+^ group. (D, E) SENP1 depletion promoted IH‐induced SUMOylation enhancement of NEMO. (D, F) SENP1 depletion decreased the level of NEMO in the hippocampus of mice. ^***^ *P* < .001, versus the SENP1^+/+^ group. (G–J) The expression of IκB‐α, IL‐1β and TNF‐α in the hippocampus of mice with IH treatment was detected by Western blot analysis. n = 3 per group. ^***^ *P* < .001 versus the SENP1^+/+^ group

After IH treatment, the expression of NF‐κB p65, IκB‐α, IL‐1β and TNF‐α in mice's hippocampus was evaluated by Western blot analysis. The results showed that NF‐κB p65 was significantly increased, and IκB‐α was significantly decreased in the IH group compared to the normoxia group (Figure 6A,B). Meanwhile, IL‐1β and TNF‐α were significantly increased in the IH group compared with that in the normoxia group (Figure 6C,D), which again confirmed that IH triggered inflammatory response in vivo. To investigate the effect of SENP1 deletion on IH‐induced inflammatory response, we knocked down the gene of SENP1 in mice using gene‐editing technology, and the genotype of mice and the knockdown efficiency of SENP1 were identified with DNA agarose electrophoresis and Western blot analysis, respectively. DNA agarose electrophoresis results showed that wild‐type mice's genotype was homozygote, and the SENP1 knockdown mice was mutant heterozygote (Figure 7A). Western blot analysis results showed that the expression of SENP1 was significantly decreased in SENP1 knockdown mice compared to that in wild‐type mice (Figure 7B,C). Besides, we also observed that the expression of SUMO‐1 was significantly increased after SENP1 knockdown (Figure 7B,C). These results suggested that SENP1 knockdown enhanced the SUMOylation in vivo. After IH treatment, we evaluated the effect of SENP1 deletion on the SUMOylation of NEMO in the hippocampus of mice with or without SENP1 knockdown by CO‐IP. The results showed that SENP1 depletion significantly promoted the SUMOylation of NEMO (Figure 7D,E) and significantly decreased the level of NEMO (Figure 7D,F), suggesting that SENP1 deletion decreased the level of NEMO in mice's hippocampus and accelerated the SUMOylation of NEMO induced by IH. Next, the expression of IκB‐α, IL‐1β and TNF‐α in mice's hippocampus was detected by Western blot analysis. The results showed that SENP1 depletion significantly decreased the expression of IκB‐α and increased the expression of IL‐1β and TNF‐α in the hippocampus of mice after IH treatment (Figure 7G–J), suggesting that SENP1 deletion up‐regulated the inflammatory response in mice's hippocampus and accelerated the SUMOylation of NEMO induced by IH.

### 3.7 Deletion of SENP1 accelerates IH‐induced cognitive dysfunction

**FIGURE 8:**
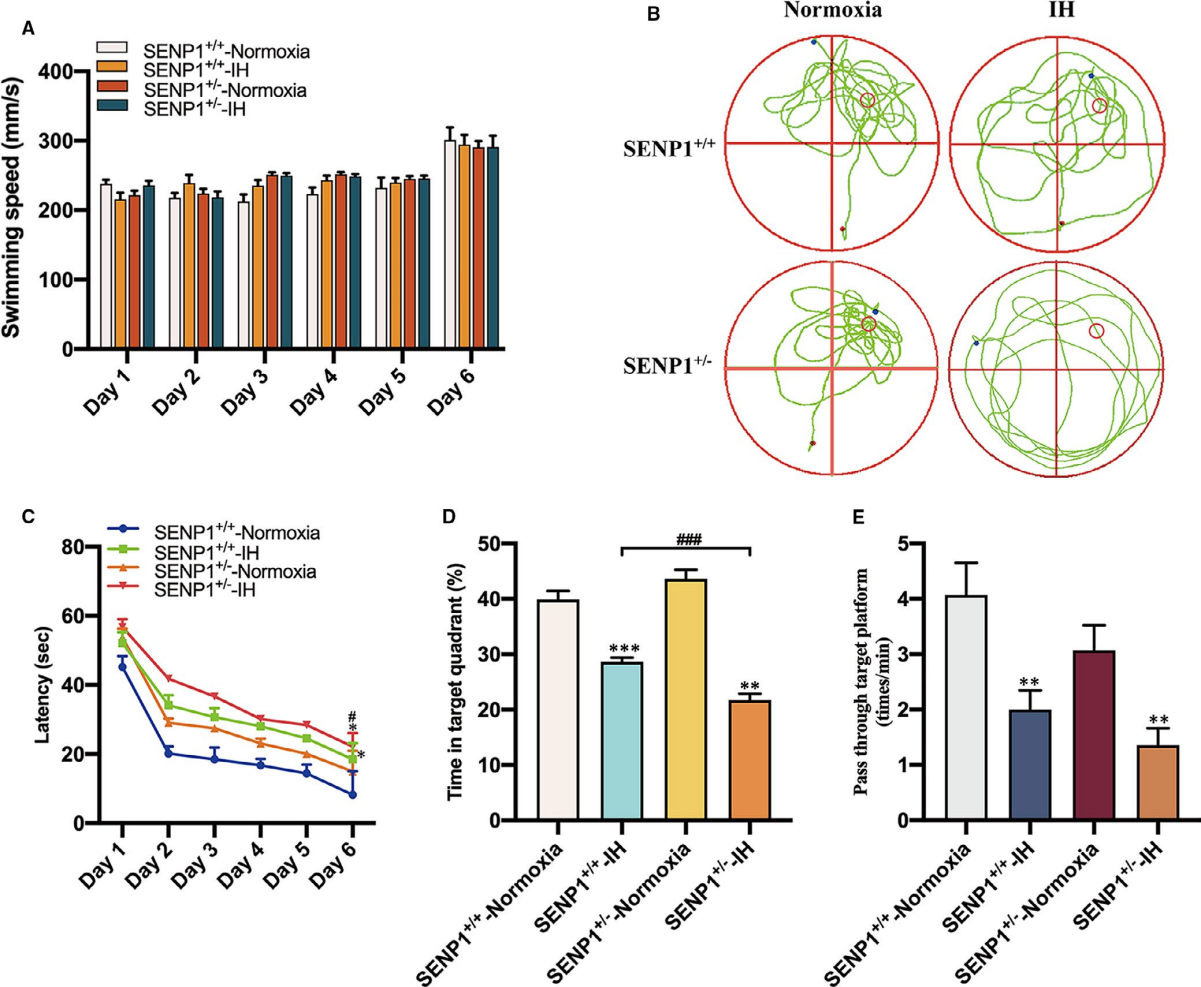
SENP1 depletion promotes IH‐induced cognitive decline. (A–E) Cognitive function of mice with or without SENP1 knockdown under normoxia and IH conditions was analysed by Morris water maze (MWM) test. Mice swim in the pool for 60 seconds at day 6, and other days’ test termination time was subject to the climbing platform. (A) There was no difference among four experimental groups in average swimming speed throughout 6 consecutive days. (B) The swimming trail map of mouse in the swimming pool during 60 seconds at day 6. (C) Latency, the time that mice first climbed onto the hidden platform or first passed through the area where the platform was located. (D) The percentage of time that mice spent in the quadrant where the platform is located versus the total time. (E) The frequency of passing through the area where the platform is located. n = 12 per group. ^*^ *P* < .05, ^**^ *P* < .01, ^***^ *P* < .001 versus the normoxia groups. **^###^** *P* < .001 versus the SENP1^+/+^ group under IH condition

Before IH treatment, mice's learning and memory abilities of spatial orientation were evaluated by the MWM test. Results showed that mice's learning and memory abilities of spatial orientation were not significantly different (*P* > .05), indicating that SENP1 depletion did not affect mice's cognitive ability under normoxic condition. After IH treatment, MWM test results showed that mice's swimming speed had no statistical difference throughout six consecutive days (*P* > .05, Figure 8A), suggesting that neither IH nor SENP1 depletion altered mice's motor ability of mice. However, the swimming track showed the mice with IH treatment spent more time in the non‐target quadrant than that with normoxia treatment, and this phenomenon is even more obvious in the SENP1 depletion group after IH treatment (Figure 8B). Besides, both IH groups exhibited longer escape latency than normoxia groups, and SENP1 depletion group exhibited much longer latency than that in the wild‐type group at day 6 (Figure 8C). Meanwhile, the percentage of time spent in the target quadrant and the frequency of passing through the target platform area were significantly reduced in both IH groups. Of note, the reductions were more distinct in the SENP1 depletion group than that in the wild‐type group (Figure 8D,E). However, there is no statistical significance in the frequency of passing through the target platform area in the SENP1 depletion group compared to the wild‐type group (Figure 8E). These data suggest that IH impaired mice's cognitive ability, and SENP1 depletion further deteriorated this impairment.

## 4 DISCUSSION

As empirical evidence implicated that SUMOylation participated in the regulation of protein stability, which promotes protein's degradation.^32^, ^33^ However, the precise mechanisms of SUMOylation in microglia involved in IH impairment remain unknown. Herein, results showed that IH induced inflammatory response in vitro and in vivo. SENP1 overexpression attenuated the inflammatory response by inhibiting the SUMOylation of NEMO after IH treatment in vitro; in contrast, SENP1 deletion promoted the inflammatory response by enhancing the SUMOylation of NEMO after IH treatment in vivo. Besides, IH induced cognitive decline in mice, and SENP1 deletion aggravated IH‐induced cognitive decline. In summary, the partial molecular mechanism of IH‐related cognitive impairment is that IH enhances the SUMOylation of NEMO and decreases the level of NEMO and the expression of IκB‐α, and de‐conjugated NF‐κB is transported into the nucleus of microglia to promote the NF‐κB‐dependent inflammatory gene transcription and then creates the inflammatory response. Finally, chronic inflammatory status results in cognitive decline.

Microglia‐mediated inflammation plays an essential role in the development and repair in brain^34^, ^35^ and is associated with IH‐related cognitive dysfunction.^36^ Activated microglia can secrete cytokines, such as IL‐1β, TNF‐α, adhesion molecules and other signalling mediators.^37^ The cytokines can further activate more microglia, leading to a vicious inflammatory cascade reaction,^38^ and this persistent chronic inflammatory status can result in neuronal damage.^39^, ^40^ Microglia can be activated as M1 and M2 phenotypes. The M1 phenotype predominates at the neuroinflammation site and accelerates the secretion of inflammatory factors. On the contrary, the M2 phenotype is responsible for tissue reconstruction by promoting the secretion of anti‐inflammatory factors.^41^, ^42^ Previous hypoxic‐ischaemic brain injury study showed that hypoxia might facilitate M1 phenotype polarization and attenuate M2 phenotype activation.^43^ Therefore, given neurological damage related to inflammation, neuroinflammation regulated by microglia polarization is an essential factor in CNS damage. Although microglia have been recognized as an important contributor to IH‐induced neuroinflammation, the specific mechanism is still unclear. Among the tiny amount of studies, one study reported that microglia‐mediated inflammation might directly result in CNS damage through TLR4 signalling induced by IH.^44^ In addition, our previous study has also indirectly confirmed that IH could activate microglia skewing to M1 phenotype, indicating the essential role of microglia in IH‐induced neurological impairment.^12^ Therefore, at the beginning of the study, the results revealed that IH significantly elevated the levels of IL‐1β and TNF‐α in microglia. IH‐induced inflammatory response of microglia was verified again.

NF‐κB pathway may implicate in the mechanism of IH‐induced neuroinflammatory response,^45^ which consists of NF‐κB 1 (p50), NF‐κB 2 (p52), RelA (p65), RelB and c‐Rel. Two separate pathways for NF‐kB activation have been verified, including canonical pathway and alternative pathway.^46^ In the canonical pathway, IKK‐β removes IκB‐α from the IκB‐α/NF‐κB complex, leading to IκB‐α degradation, and NF‐κB is freed and then transported to the nucleus to promote the NF‐κB‐dependent gene transcription,^46^ thus inducing the deposition of NF‐κB in the nucleus. Therefore, the reduction of IκB‐α can indirectly reflect the activation of NF‐κB in the canonical pathway.^47^ The canonical pathway can be activated by proinflammatory cytokines such as TNF‐α and IL‐1, resulting in the activation of RelA‐ or cRel‐ containing complexes. Meanwhile, TNF family cytokines and B cell activating factor can activate the alternative pathway.^47^ Moreover, previous study has proved that hypoxia primarily activates NF‐κB pathway through the canonical pathway.^48^ Besides, our previous study showed that IH could induce the release of proinflammatory cytokines from microglia via the NF‐κB/p38 MAPK pathway.^12^ In the present study, the in vitro and in vivo results showed IH inhibited the expression of IκB‐α, indicating IH probably activated NF‐κB pathway in the canonical pathway, and was also confirmed by the up‐regulated expression of IL‐1β and TNF‐α. However, the specific mechanism of IH activated NF‐κB pathway in microglia remains unclear.

SUMOylation is extensively participated in the modification of the essential protein in a variety of cellular processes such as signal transduction, transcription, nuclear transport and genome integrity.^20^, ^33^, ^49^ SUMOylation is a dynamic and reversible process that can be catalysed by SUMO‐specific activating, conjugating and ligating enzymes.^18^ The reversal of SUMOylation is conducted by SUMO‐specific proteases (SENPs) family. SENP1 was the first identified SUMO‐specific protease and participated in various diseases.^20^, ^21^, ^26^ For example, SENP1 deletion destroyed carcinoma cells’ growth, migration and invasion, and enhanced its apoptosis in hepatocellular carcinoma.^23^ Besides, the expression of SENP1 in precancerous prostatic tissue was significantly up‐regulated compared to the adjacent normal prostate epithelia,^20^ indicating that SENP1 could regulate the development of tumours. Importantly, evidence has been reported that SENP1 deletion promotes the SUMOylation of NEMO in adipocytes, thus activating NF‐κB pathway, cytokines generation and pancreatic inflammatory response,^26^ suggesting that SENP1 could activate NF‐κB pathway through the de‐SUMOylation of NEMO. Herein, the mechanism of IH‐induced inflammatory response through the de‐SUMOylation of NEMO by SENP1 in microglia was elucidated. Previous study showed that the SUMOylation of NF‐κB p65 could enhance the nuclear import of NF‐κB p65 and the activation of NF‐κB.^50^ Herein, our results exhibited that IH only enhanced the SUMOylation of NEMO, not NF‐κB p65, and decreased the expression of SENP1, indicating that IH activated the NF‐κB pathway through the SUMOylation of NEMO.

Our previous study showed that SENP1 overexpression could suppress IH‐induced inflammatory response in microglia, as evidence of the reduction of TNF‐α and IL‐6 in vitro.^25^ In the present study, IH reduced the expression of SENP1 was verified again in vitro and in vivo. Accordingly, after the overexpression of SENP1 in BV‐2 cells, the co‐immunoprecipitation (CO‐IP) results exhibited that SENP1 significantly inhibited SUMOylation of NEMO and enhanced the level of NEMO induced by IH in vitro. Moreover, SENP1 deletion significantly promoted IH‐induced SUMOylation of NEMO, thus reduced the level of NEMO in the hippocampus of mice. These results confirmed the regulatory ability of SENP1 to de‐SUMOylated NEMO and the expression of NEMO. Similarly, the down‐regulated expression of IκB‐α and the enhancement expression of IL‐1β and TNF‐α induced by IH were also reversed by SENP1 overexpression in vitro. In contrast, these alterations were promoted by SENP1 knockdown in vivo. After the overexpression of SENP1, the inhibition of IH‐induced inflammatory response was abolished by siRNA‐NEMO. Collectively, SENP1 could regulate IH‐induced inflammatory response in microglia through NF‐κB pathway, primarily regulating the SUMOylation of NEMO, affecting the expression of IκB‐α. As previous study illustrated that SENP1 played a protective role in brain ischaemia.^51^ Hippocampus is responsible for learning and memory and particularly vulnerable to excessive inflammatory response.^52^ Therefore, we took the hippocampus as our target in this study. In addition, the mechanism between IH‐induced inflammation and cognitive decline has been rarely studied in OSA and was mainly focussed on the influence of IH on Alzheimer's disease or postoperative cognitive dysfunction. Therefore, further clinical and basic studies are required to investigate the specific mechanism involved in the pathogenesis of OSA‐mediated brain structural damages and cognitive alteration. So, we investigated the mechanism of IH associated cognitive decline, which revealed that SENP1 played an anti‐inflammatory role in IH‐induced inflammatory response and a protective role in the hippocampus of IH impairment. More importantly, SENP1 overexpression inhibited the IH‐induced inflammatory response through NF‐κB pathway by accelerating the de‐SUMOylation of NEMO in vitro, and SENP1 depletion promoted the IH‐induced inflammatory response through NF‐κB pathway by enhancing the SUMOylation of NEMO in vivo. So, we evaluated cognitive function of mice after IH treatment by MWM test, which demonstrated that SENP1 depletion seriously aggravated IH‐induced cognition decline with the evidence of increased escape latency, decreased percentage of time spent in the target quadrant and the frequency of passing through the target platform area in mice with SENP1 knockdown after IH treatment, which suggested that SENP1 plays essential roles in anti‐inflammation and protective effect in IH‐induced cognitive impairment.

In conclusion, we highlighted a novel mechanism of SENP1 on microglia‐mediated neuroinflammatory response during IH‐induced cognitive dysfunction. We also provided evidence on the mechanism that SENP1 regulates microglia‐mediated inflammatory response through the de‐SUMOylation of NEMO, leading to the inhibition of NF‐κB signalling pathway, and supports the future gene or other basic biological therapy of IH‐related cognitive dysfunction in OSA.

## CONFLICT OF INTERESTS

All authors declare that have no conflict of interests.

## AUTHOR CONTRIBUTION

**Hongwei Wang:** Data curation (equal); Formal analysis (equal); Methodology (equal); Software (equal); Validation (equal); Visualization (equal); Writing‐original draft (equal). **Tianyun Yang:** Formal analysis (equal); Software (equal); Validation (equal). **Jinyuan Sun:** Data curation (equal); Methodology (equal). **Sisen Zhang:** Methodology (equal); Supervision (equal); Writing‐review & editing (equal). **Song Liu:** Conceptualization (equal); Funding acquisition (equal); Project administration (equal); Resources (equal); Supervision (equal); Writing‐review & editing (equal).

## Data Availability

The data used to support the findings of this study are included in the article.
